# Were Eli Lilly Unaware of This Study?

**DOI:** 10.1371/journal.pmed.0030408

**Published:** 2006-09-26

**Authors:** Aasa Reidak

The authors of this study [1] claim that no competing interests exist. However, Julio Licinio presented a talk at Lilly Research Laboratory in Indianapolis, Indiana, on March 16, 2005, on “Depression, Antidepressants, and Suicidality: A Critical Appraisal” and “Suicide in the U.S. 1960–2002: Impact of Fluoxetine Prescriptions”, slightly over a year before the study was published. This is noted on page 19 of Dr. Licinio’s 51 page curriculum vitae [2]. In a *Medical News Today* article [3], Eli Lilly claims to not have known about this study until it was accepted for publication. I don’t see how they could not have known about Dr. Licinio’s study, when he presented on the very topic at Eli Lilly Research Laboratory in Indianapolis on March 16, 2005.
